# Recent advances in total synthesis of illisimonin A

**DOI:** 10.3762/bjoc.21.199

**Published:** 2025-11-20

**Authors:** Juan Huang, Ming Yang

**Affiliations:** 1 State Key Laboratory of Natural Product Chemistry, College of Chemistry and Chemical Engineering, Lanzhou University, 222 South Tianshui Road, Lanzhou, Gansu Province 730000, China

**Keywords:** bioinspired synthesis, biosynthetic pathway, *Illicium* sesquiterpene, illisimonin A, total synthesis

## Abstract

*Illicium* sesquiterpenes are a large class of highly oxygenated and sterically congested sesquiterpenoids isolated from the genus *Illicium*. Illisimonin A stands out as one of the most structurally intricate members of this family, featuring a novel bridged tricyclo[5.2.1.0^1,5^]decane carbon framework designated as the “illisimonane” skeleton. This core ring system is further embellished by additional bridging via a γ-lactone and a γ-lactol ring, resulting in a caged pentacyclic scaffold with a 5/5/5/5/5 ring arrangement. The compound demonstrates neuroprotective activity by mitigating oxygen-glucose deprivation-induced cell injury in SH-SY5Y cells. Since its isolation in 2017, illisimonin A has garnered significant interest from the synthetic chemistry community. To date, five research groups have accomplished the total synthesis of illisimonin A. This review offers a comprehensive overview of its isolation, proposed biosynthetic pathway and the synthetic strategies employed in its total synthesis.

## Introduction

**Figure 1:**
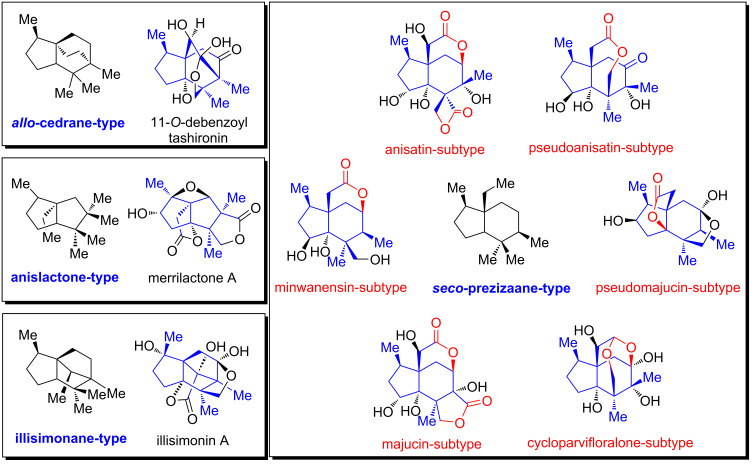
The categorization of *Illicium* sesquiterpenes and representative natural products.

The genus *Illicium*, the sole member of the family Illiciaceae, is a rich source of sesquiterpenoid natural products. To date, a wide variety of sesquiterpenes have been isolated from this genus, among which *Illicium* sesquiterpenes represent a prominent group. Since the first isolation of anisatin in 1952 [1], more than 100 *Illicium* sesquiterpenes have been isolated from over 40 species of *Illicium* [2]. Based on their carbon skeletons, they can be classified into the following types: *allo*-cedrane, anislactone, *seco*-prezizaane and illisimonane (Figure 1). The illismonane-type is the most recent identified. The *seco*-prezizaane-type can be further divided into six subtypes according to their lactone patterns, namely anisatin-subtype, pseudoanisatin-subtype, pseudomajucin-subtype, cycloparvifloralone-subtype, majucin-subtype, and miwanensin-subtype (Figure 1). The seminal work by the Fukuyama group demonstrated that some of these natural products exhibit potent neurite outgrowth-promoting activity in primary cultured rat cortical neurons, which has attracted considerable interest from synthetic chemists. Although the intricate structures of this family have posed significant challenges to chemical synthesis, more than 30 total syntheses of *Illicium* sesquiterpenes have been reported until now [3–25].

**Figure 2:**
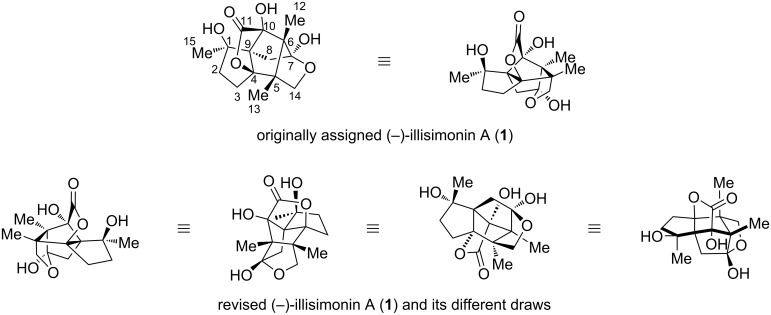
The original assigned (−)-illisimonin A, revised (−)-illisimonin A, and their different draws.

In 2017, Yu and co-workers isolated a new *Illicium* sesquiterpene, namely illisimonin A, from the fruits of *Illicium simonsii* [26]. Unlike other *Illicium* sesquiterpenes, illisimonin A features an unprecedented bridged tricyclo[5.2.1.0^1,5^]decane carbon framework that incorporates a highly strained *trans*-pentalene subunit. This carbon ring system is further bridged with a γ-lactone and a γ-lactol ring, forming a caged pentacyclic scaffold with a 5/5/5/5/5 ring arrangement. Illisimonin A was thus classified as an illisimonane-type *Illicium* sesquiterpene, and its carbon skeleton was designated as “illisimonane skeleton”. The absolute configuration of (−)-illisimonin A was determined to be 1*R*,4*R*,5*R*,6*R*,7*S*,9*S*,10*S* by comparing the calculated electronic circular dichroism (EDC) spectrum with experimental CD data (Figure 2). Biological evaluation revealed that illisimonin A exhibits neuroprotective effects against oxygen-glucose deprivation-induced cell injury in SH-SY5Y cells, suggesting its potential as a lead compound for the treatment of neurodegenerative diseases.

**Scheme 1:**
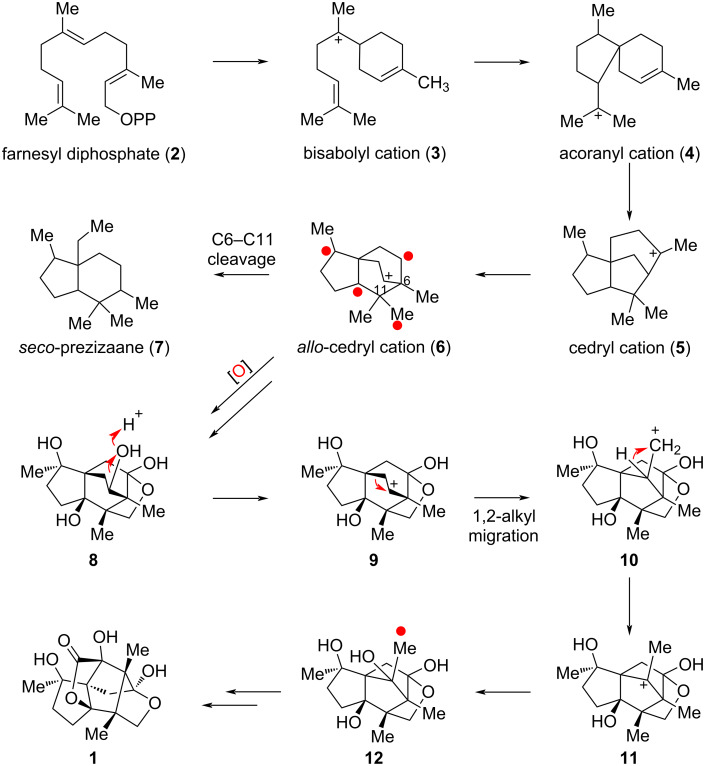
Proposed biosynthetic pathway of illisimonin A by Yu et al.

The possible biosynthetic pathway of illisimonin A was also proposed by Yu and co-workers, as illustrated in Scheme 1. The proposed biosynthetic pathway clarifies the relationship between illisimonin A and other *Illicium* sesquiterpenes. *Allo*-cedrane-type, *seco*-prezizaane-type and illismonane-type *Illicium* sesquiterpenes are all biosynthesized from farnesyl diphosphate (**2**) through a series of cationic cyclizations and migrations. The 5/6/6 tricarbocyclic *allo*-cedrane framework **6** serves as the key biogenetic intermediate for both the *seco*-prezizaane and illismonane skeletons. The conversion of the *allo*-cedrane skeleton to the illismonane skeleton was hypothesized to proceed via a 1,2-alkyl migration of intermediate **9** to **10**. However, subsequent density functional theory (DFT) calculations by the Tantillo group on rearrangements of potential biosynthetic precursors revealed that structure **10** corresponds to a transition state rather than a stable intermediate of the 1,2-alkyl migration [27]. Their study further indicated that only certain precursors with certain specific oxidation patterns are competent to undergo this rearrangement.

The same as other *Illicium* sesquiterpenes, the highly oxgenated and strained skeleton of illisimonin A has posed a significant challenge to synthetic chemists. To date, the research groups of Rychnovsky [28], Kalesse [29], Yang [30], Dai [31] and Lu [32] have achieved the total synthesis of this molecule. Notable, Rychnovsky and co-workers revised the absolute configuration of (−)-illisimonin A to 1*S*,4*S*,5*S*,6*S*,7*R*,9*R*,10*R*. This review summarizes the reported synthetic routes toward illisimonin A, including uncompleted approaches.

## Review

### Rychnovsky’s synthesis and the absolute configuration revision of (−)-illisimonin A

In 2019, Rychnovsky’s group reported the first total synthesis of illisimonin A [28]. Recognizing that the strained *trans*-pentalene moiety in the molecule is challenging to construct directly, the team adopted a strategy involving rearrangement from the more accessible *cis*-pentalene isomer. They first assembled the *cis*-pentalene core through an elegant intramolecular Diels–Alder (IMDA) reaction. Subsequently, the conversion from *cis* to *trans*-pentalene was achieved via a semipinacol rearrangement. Finally, a White–Chen C–H oxidation [33–35] was employed to install the lactone ring, thereby completing the synthesis.

**Scheme 2:**
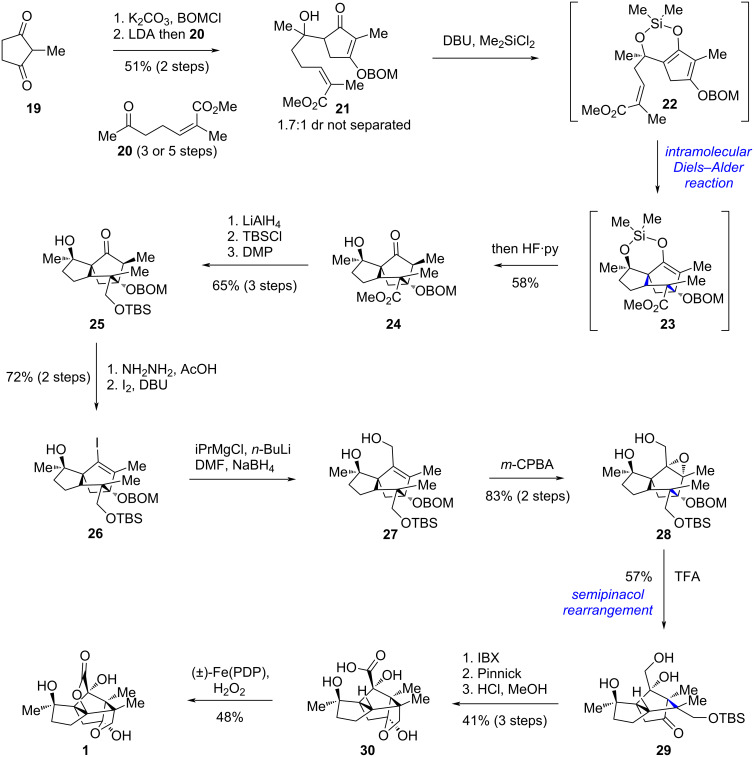
Rychnovsky’s racemic synthesis of illisimonin A (**1**).

The synthesis began with commercially available compound **19** and known compound **20** (Scheme 2). These were joined via an intermolecular aldol reaction to give adduct **21**, obtained as a 1.7:1 mixture of diastereomers after protection of one of the carbonyl groups in **19** as enol ether with BOMCl. A silyl-tethered intramolecular Diels–Alder reaction of the in situ generated **22** constructed the tricyclo[5.2.1.0^1,5^]decane core bearing a *cis*-pentalene unit, yielding compound **23**, which was subsequently subjected to a one-pot desilylation to afford **24**. Reduction of both the ester and ketone functionalities in **24**, followed by selective protection of the primary alcohol and re-oxidation of the secondary alcohol to ketone, furnished compound **25** in three steps. The ketone in **25** was then converted to vinyl iodide **26** via hydrazine formation followed by iodination using Barton’s method. Subsequent Bouvealt aldehyde synthesis and in situ reduction delivered allylic alcohol **27**. Epoxidation of **27** with *m*-CPBA afforded the rearrangement precursor **28**. Protonic acid-promoted semipinacol rearrangement of **28** enabled the rearrangement of *cis*-pentalene to *trans*-pentalene, delivering intermediate **29**, which possesses the same carbon skeleton as the natural product. Further oxidation of the primary alcohol to a carboxylic acid, accompanied by TBS deprotection, afforded hemiketal **30**. Finally, a White−Chen C–H oxidation [33–35] of **30** installed the lactone, completing the synthesis of racemic illisimonin A (**1**).

**Scheme 3:**
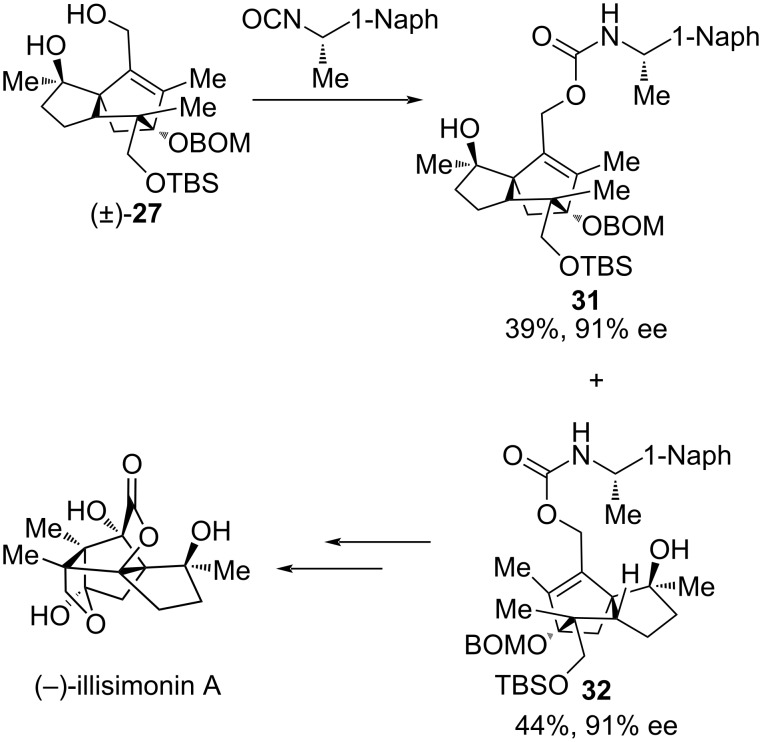
The absolute configuration revision of (−)-illisimonin A.

Noting that the C1 configuration of illisimonin A was opposite to that of other *Illicium* sesquiterpenes, Rychnovsky’s group sought to confirm the absolute configuration of the natural product. They resolved racemic intermediate **27** by derivatization with (*S*)-1-(1-naphthyl)ethyl isocyanate, followed by separation of the resulting diastereomers via silica gel chromatography (Scheme 3). By converting diastereomer **32** to (−)-illisimonin A, the absolute configuration of the natural product was conclusively revised to 1*S*,4*S*,5*S*,6*S*,7*R*,9*R*,10*R*.

### Kalesse’s asymmetric synthesis of illisimonin A

In 2023, Kalesse and co-workers reported an asymmetric synthetic route to illisimonin A [29]. The Kalesse group also noticed the strained *trans*-pentalene in illisimonin A. Since there is a spiro substructure hidden inside the natural product’s cage-like ring system, Kalesse’s group chose to construct this architecture first using a tandem Nazarov/ene cyclization [36]. The *cis*-pentalene was subsequently assembled via a Ti(III)-mediated epoxide–ketone coupling reaction.

**Scheme 4:**
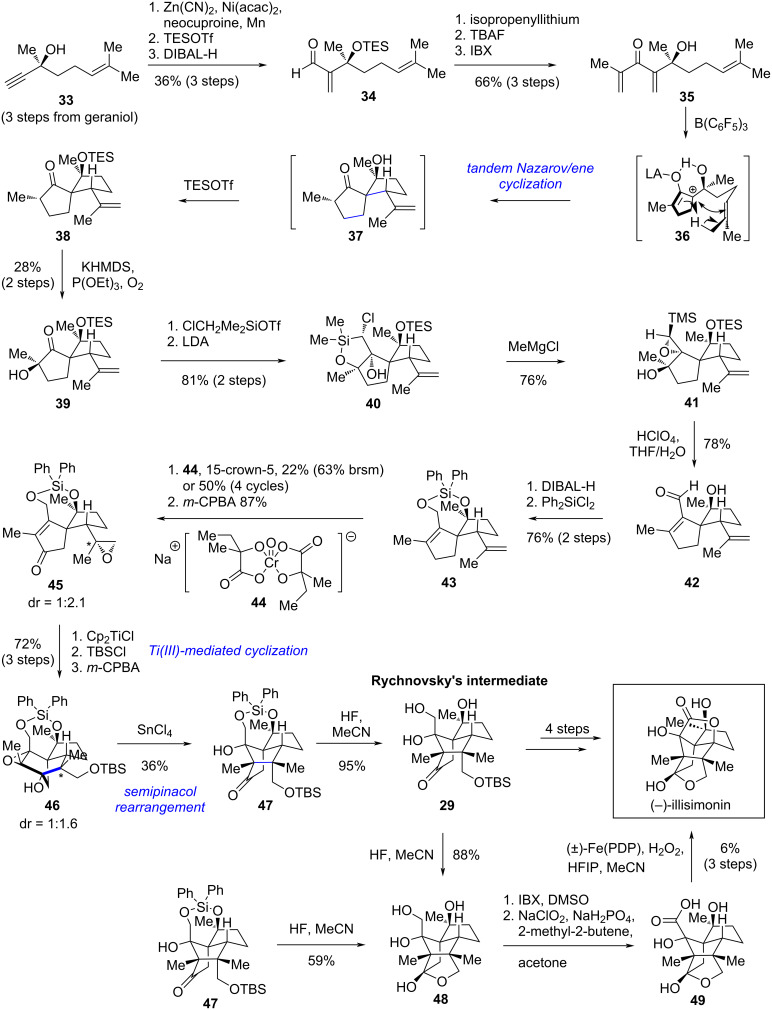
Kalesse’s asymmetric synthesis of (−)-illisimonin A.

Starting from the known enantioenriched compound **33**, a nickel-catalyzed hydrocyanation of the terminal alkyne was performed. Subsequent protection of the tertiary alcohol with TESOTf and reduction of the resulting cyanide to an aldehyde afforded compound **34** (Scheme 4). Addition of isopropenyllithium to aldehyde **34**, followed by TES deprotection and oxidation of the secondary alcohol, yielded the cyclization precursor **35**. A B(C_6_F_5_)_3_-catalyzed tandem Nazarov/ene cyclization of **35** provided the key spirocyclic intermediate **37**. The tertiary alcohol was protected in situ with TESOTf to suppress retro-aldol side reactions. Notably, prior TES deprotection of the cyclization precursor was essential, as the TES-protected analogue of **35** failed to deliver the desired spirocycle **38** under the Nazarov cyclization conditions. α-Oxidation of **38** with molecular oxygen afforded **39**, which was then converted to **40** via formation of a chloromethyl silyl ether, deprotonation, and intramolecular addition to ketone. Treatment of the silacycle with MeMgCl cleaved the Si–O bond and subsequent intramolecular nucleophilic substitution of the chloride with the adjacent hydroxy group yielded TMS-epoxide **41**. Protonic acid-mediated opening of the TMS-epoxide, accompanied by TES deprotection, afforded enal **42**. To avoid the chemoselectivity issues in the subsequent allylic oxidation and radical cyclization steps, enal **42** was converted to **43** by reduction of the aldehyde and protection of the resultant diol with Ph_2_SiCl_2_. Allylic oxidation of **43** with **44** [37] afforded the enone in 22% yield (63% brsm) or 50% yield after four cycles with recovery of starting material. Selective epoxidation of the isopropenyl group with *m*-CPBA delivered cyclization precursor **45** as an inseparable mixture of diastereomers (dr = 1:2.1). A Cp_2_TiCl-mediated cyclization of **45** constructed the tricyclo[5.2.1.0^1,5^]decane core with a *cis*-pentalene unit. The product was further processed into rearrangement precursor **46** (as an inseparable mixture, dr = 1:1.6) by TBS protection of the primary alcohol and epoxidation of the alkene with *m*-CPBA. Unlike Rychnovsky’s substrate, epoxy alcohol **46** underwent rearrangement only under Lewis acidic conditions to furnish **47**. Selective TES deprotection with HF afforded Rychnovsky’s intermediate **29**. (−)-Illisimonin A was obtained in 13% yield over the same 4-step sequence as reported by Rychnovsky’s group. An alternative 4-step endgame starting from **47** was also developed, albeit with a lower overall yield of 3%.

### Yang’s bioinspired synthesis of illisimonin A

**Scheme 5:**
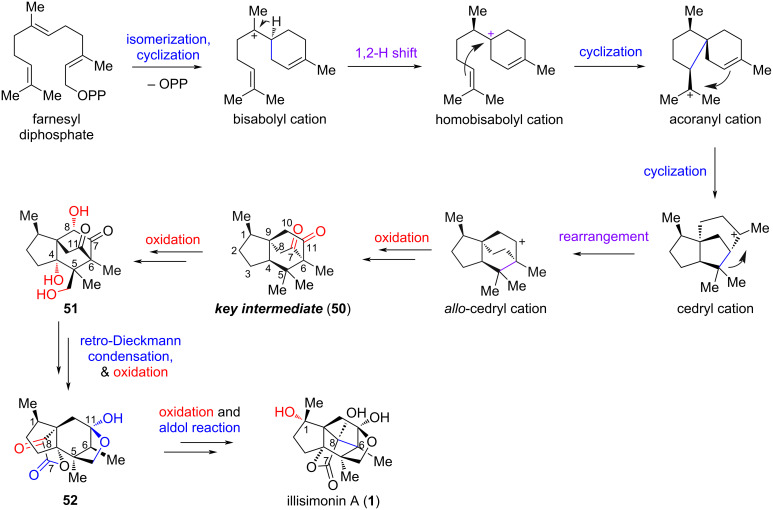
Yang group proposed biosynthetic pathway of illisimonin A.

In 2023, Yang and co-workers reported a bioinspired divergent synthesis of illisimonin A and merrilactone A, which belonged to illisimonane-type and anislactone-type *Illicium* sesquiterpenes, respectively [30]. They proposed that a deeper understanding of the biosynthetic pathway of *Illicium* sesquiterpenes could facilitate a divergent total synthesis of this family of natural products, even among members with distinct carbon skeletons. Since previously proposed biosynthetic pathways lacked key mechanistic details – particularly the critical reactions responsible for skeletal diversity – Yang and co-workers first introduced a comprehensive and detailed biosynthetic pathway for *Illicium* sesquiterpenes, with the route to illisimonin A depicted in Scheme 5. The transformations from farnesyl diphosphate to the *allo*-cedryl cation were consistent with earlier reports [2,26], though the configurations of the intermediates were clearly delineated. The authors proposed that dicarbonyl compound **50** serves as the key intermediate diverging to all *Illicium* sesquiterpenes, with a retro-Dieckmann condensation and aldol reaction identified as the key steps enabling the transformation from the *allo*-cedrane skeleton to the illisimonane framework.

**Scheme 6:**
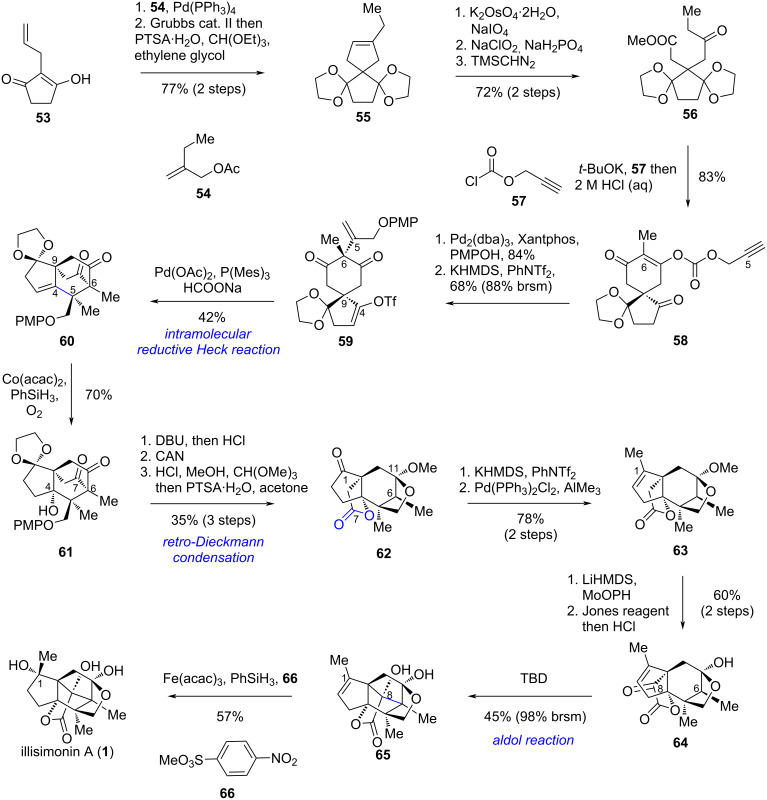
Yang’s bioinspired synthesis of illisimonin A.

Inspired by the proposed biosynthetic pathway, Yang et al. designed a synthetic route as shown in Scheme 6. Starting from the known compound **53**, a Tsuji–Trost allylation was employed to introduce another side chain, affording a diene intermediate. A subsequent ring-closing metathesis (RCM) reaction formed the cyclopentene ring, and one pot protection of both carbonyl groups with ethylene glycol provided bis-ketal **55**. Notably, due to steric hindrance, only one carbonyl group could be protected prior to the RCM step. Oxidative cleavage of the cyclopentene followed by Pinnick oxidation of the resulting aldehyde to the carboxylic acid and esterification yielded ketoester **56**. Dieckmann condensation of **56**, esterification of the resulting enolate with **57**, and subsequent one-pot partial deketalization afforded carbonate **58**. A palladium-catalyzed decarboxylative alkenylation reaction was then carried out across the less hindered face of the six-membered ring to connect C5 and C6. Selective deprotonation and triflation at the C4 carbonyl group provided enol triflate **59**. An intramolecular reductive Heck reaction of **59** enabled the transannular connection between C4 and C5, generating key intermediate **60**, which possesses the same carbon skeleton as the proposed biosynthetic key intermediate **50** and contains the suitable functional groups for further elaboration. Mukaiyama hydration of **60** introduced a tertiary alcohol at the C4 position, yielding retro-Dieckmann precursor **61**. Subsequent retro-Dieckmann condensation under basic conditions, deprotection of the PMP group, and selective ketalization of the C11 carbonyl group afforded compound **62**. The C1 methyl group was installed via enol triflate formation followed by a palladium-catalyzed coupling reaction with AlMe_3_. The carbonyl group at C8, required for the subsequent aldol reaction, was introduced by enolate oxidation followed by Jones oxidation. Hydrolysis of the ketal at C11 afforded ketoester **64**. A TBD (1,5,7-triazabicyclo[4.4.0]dec-5-ene)-catalyzed intramolecular aldol reaction connected C6 and C8, assembling the *trans*-pentalene ring and affording the core carbon framework of illisimonin A. The C1 hydroxy group was initially introduced by a Mukaiyama hydration reaction using O_2_ as the stoichiometric oxidant; however, illisimonin A was obtained only as a minor product. When O_2_ was replaced with the nitroaromatic compound **66** [38], the diastereoselectivity was reversed, thereby providing illisimonin A in 57% yield as a single diastereomer. The authors proposed that a hydrogen bond between the nitro group of **66** and the C8 hydroxy group could be responsible for this reversal in selectivity.

### Dai’s asymmetric synthesis of (−)-illisimonin A

In 2025, Dai and co-workers accomplished an asymmetric total synthesis of (−)-illisimonin A in 16 steps from (*S*)-carvone (**67**) using a pattern-recognition strategy and five sequential olefin transpositions [31].

**Scheme 7:**
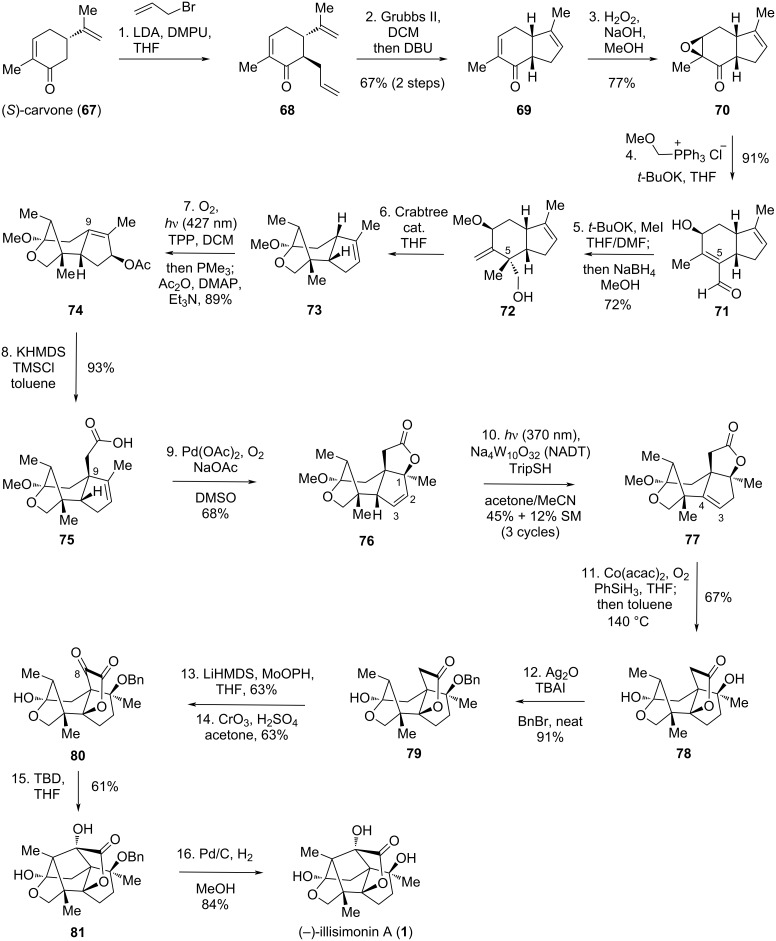
Dai’s asymmetric synthesis of (–)-illisimonin A.

Starting from (*S*)-carvone (**67**), reaction with allyl bromide introduced an allyl group to give **68**, which was then converted to the bicyclic compound **69** via a ring-closing metathesis (RCM) reaction followed by one-pot epimerization at the α-position of the carbonyl group (Scheme 7). Chemoselective epoxidation of the enone double bond in **69** yielded epoxide **70**. A Wittig reaction of **70** with (methoxymethyl)triphenylphosphonium chloride and *t*-BuOK generated a methyl enol ether, which was unstable in the presence of the epoxide. During aqueous workup, simultaneous hydrolysis of the enol ether and epoxide ring-opening afforded **71**. To install the all-carbon quaternary center at C5, compound **71** was treated with *t*-BuOK and MeI, enabling the deprotonation of the α,β-unsaturated aldehyde and methylation at C5; this step also facilitated protection of the secondary alcohol. The aldehyde was reduced in the same pot to give **72**. Isomerization of the allylic methyl ether to an enol methyl ether was achieved using Crabtree’s catalyst in refluxing THF. Subsequent ketalization with the primary alcohol yielded the bridged ketal **73**. A Schenck ene reaction on **73** induced the second olefin isomerization, generating an allylic alcohol that was acetylated in situ to provide **74**. An Ireland–Claisen rearrangement facilitated the third olefin transposition, concurrently forming an all-carbon quaternary center at C9 and affording carboxylic acid **75**. The fourth olefin transposition was achieved via a palladium-catalyzed oxidative lactonization, yielding **76** with a newly established quaternary center at C1 and isomerization of the double bond to C2=C3. Photocatalyzed isomerization of the C2–C3 double bond in **76** to C3=C4 furnished **77** [39–40]. A Mukaiyama hydration introduced a hydroxy group at C4, accompanied by *trans*-esterification to give lactone **78**. The resulting tertiary alcohol was protected as its benzyl ether to afford **79**. A two-step oxidation protocol, analogous to Yang’s method, introduced the C8 carbonyl group, yielding **80**. The final ring was closed via an intramolecular aldol reaction following Yang’s conditions [30], assembling the *trans*-pentalene to give **81**. Finally, deprotection of the benzyl ether delivered (−)-illisimonin A (**1**).

### Lu’s gram-scale synthesis of illisimonin A

In 2025, the Lu group reported a gram-scale total synthesis of illisimonin A in 15 steps from commercially available starting materials [32]. This synthesis features a pentafulvene-based intramolecular [6 + 2] cycloaddition [41–42] and a nitroso-Diels–Alder reaction [43] as key steps.

**Scheme 8:**
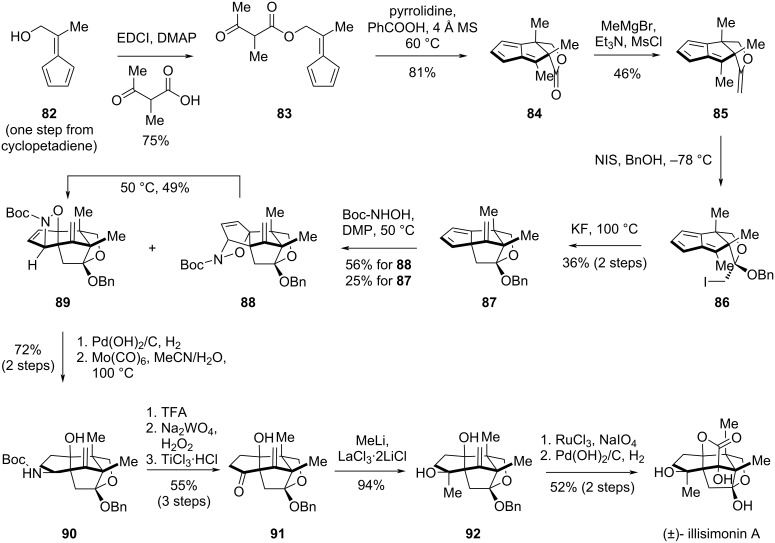
Lu’s total synthesis of illisimonin A.

The route began with the esterification of pentafulvenol **82** to give β-ketoester **83**, which was subsequently converted to the sterically encumbered tricyclic lactone **84** via an intramolecular [6 + 2] cycloaddition (Scheme 8). Attempts to achieve an asymmetric version of the cycloaddition were unsuccessful. Treatment of the lactone with MeMgBr, followed by mesylation and elimination of the resulting hemiacetal, afforded enol ether **85**. Reaction of **85** with iodine and BnOH enabled the intermolecular iodoetherification to yield ketal **86**. A KF-promoted intramolecular alkylation of the cyclopentadiene moiety then delivered compound **87**. To introduce the C4 hydroxy group and C1 functional handle for further elaboration, a nitroso-Diels–Alder reaction of **87** was employed, generating both the kinetic product **88** and the desired thermodynamic product **89**. Heating **88** promoted a retro-Diels–Alder/Diels–Alder equilibrium, favoring the more stable isomer **89**. Palladium-catalyzed hydrogenation of the 1,2-disubstituted alkene in **89**, followed by Mo(CO)_6_-mediated N–O bond cleavage afforded carbamate **90**. The carbamate was converted to a carbonyl group via Boc deprotection with TFA, oxidation of the resulting amine to the oxime with Na_2_WO_4_ and H_2_O_2_, and subsequent reduction with TiCl_3_·HCl to give **91**. The LaCl_3_·2LiCl-mediated methyl addition to the carbonyl group installed the tertiary alcohol at C1, yielding intermediate **92**. The α-hydroxy lactone was constructed through RuO_4_-mediated oxidation, forming the pentacyclic core. Finally, debenzylation of the resulting pentacyclic compound under palladium-catalyzed hydrogenation provided (±)-illisimonin A. Notably, the authors were able to obtain 1.8 g of the natural product in a single run.

**Scheme 9:**
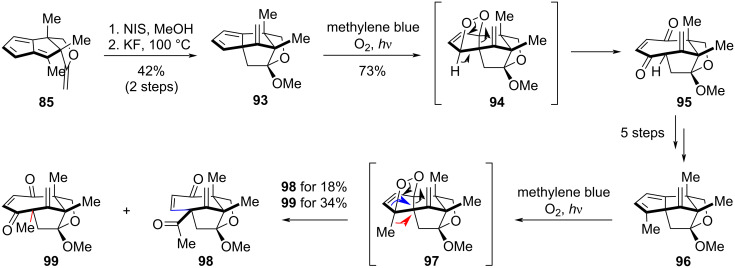
Initial efforts toward the total synthesis of illisimonin A by the Lu Group.

The successful total synthesis of illisimonin A by the Lu group was preceded by instructive setbacks, primarily in constructing the *trans*-fused 5/5 ring system. As depicted in Scheme 9, compound **85** was first transformed into the pentacyclic diene intermediate **93** via a two-step sequence. Subsequent [4 + 2] cycloaddition of **93** with singlet oxygen yielded an unstable endoperoxide adduct **94**, which rearranged to diketone **95**. A five-step sequence, featuring an intramolecular aldol reaction to assemble the pentacyclic core and the installation of the C1 methyl group, then afforded compound **96**. However, subjecting **96** to the singlet oxygen cycloaddition again led to rearrangement, producing diketones **98** and **99**. The solution was found by employing a nitroso-Diels–Alder reaction with dienophile **87**, which provided a stable adduct and ultimately enabled the completion of the synthesis. This strategic pivot offers a key lesson for handling fragile cycloaddition adducts.

### Suzuki’s synthetic effort towards illisimonin A

A synthetic study aimed at constructing the tricyclo[5.2.1.0^1,5^]decane core of illisimonin A was reported by Suzuki and co-workers in 2021 [44]. Their work proposed that this core structure could be generated from a highly oxidized *allo*-cedrane moiety through a tandem retro-Claisen/aldol reaction.

**Scheme 10:**
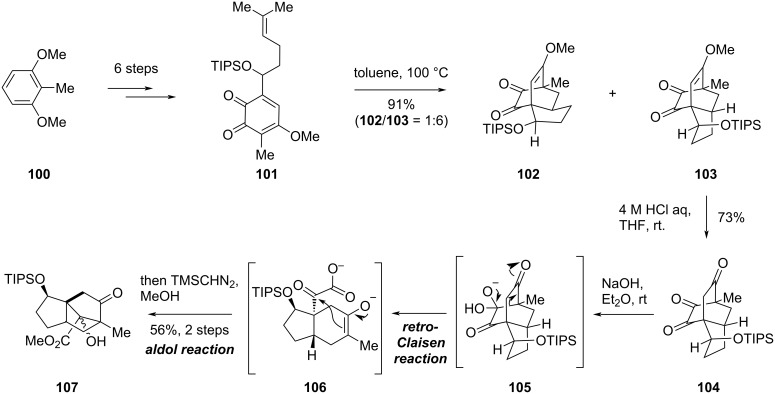
Suzuki’s synthetic effort towards illisimonin A.

Beginning with compound **100**, a 6-step sequence afforded *ortho*-quinone **101** (Scheme 10). Heating **101** promoted an intramolecular Diels–Alder reaction, affording **102** and **103** in 91% yield with a 1:6 ratio. The major product **103** was selected to investigate the tandem retro-Claisen/aldol reaction. Hydrolysis of the enol methyl ether in **103** under acidic conditions delivered triketone **104**. Subsequent treatment of **104** with aqueous NaOH facilitated a retro-Claisen reaction, yielding the intermediate **106**, which subsequently underwent an intramolecular aldol reaction to form the five-membered ring. The resulting carboxylic acid was then esterified with TMSCHN_2_ to furnish ester **107**, which possesses the characteristic tricyclo[5.2.1.0^1,5^]decane core of illisimonin A.

## Conclusion

Over the past seventy years, ongoing chemical investigations of the *Illicium* species have led to the discovery of a great number of *Illicium* sesquiterpenes. The sterically congested and highly oxygenated skeleton of *allo*-cedrane-type, anislactone-type, and *seco*-prezizaane-type *Illicium* sesquiterpenes have attracted significant interest from synthetic chemists, resulting in numerous elegant total syntheses of molecules within this family. The recent identification of illisimonin A has further expanded the structural diversity of *Illicium* sesquiterpenes. Its tricyclo[5.2.1.0^1,5^]decane core, which contains a strained *trans*-pentalene subunit, presents new synthetic challenges. A breakthrough in the total synthesis of illisimonin A was achieved by Rychnovsky and co-workers through a rearrangement strategy that also led to the correction of its absolute configuration. Kalesse and co-workers accomplished the first asymmetric total synthesis of (−)-illisimonin A based on strategies involving spiro substructure assembly and rearrangement. Consideration of the biosynthetic pathway of illisimonin A inspired Yang and co-workers to develop a bioinspired synthetic route. Employing a pattern-recognition strategy, Dai and co-workers achieved the second asymmetric total synthesis of (−)-illisimonin A in 16 steps. Lu and co-workers realized the shortest and gram-scale total synthesis of racemic illisimonin A in 15 steps by leveraging a higher-order cycloaddition. Although various synthetic strategies have been developed, only two of them are asymmetric. Designing a more efficient and asymmetric synthetic route remains a worthwhile pursuit.

## Data Availability

Data sharing is not applicable as no new data was generated or analyzed in this study.
